# PET/CT-guided percutaneous biopsy of FDG-avid metastatic bone lesions in patients with advanced lung cancer: a safe and effective technique

**DOI:** 10.1007/s00259-016-3455-9

**Published:** 2016-07-22

**Authors:** Wei Guo, Bing Hao, Hao-jun Chen, Long Zhao, Zuo-ming Luo, Hua Wu, Long Sun

**Affiliations:** Department of Nuclear Medicine & Minnan PET Center, Xiamen Cancer Hospital, The First Affiliated Hospital of Xiamen University, 55 Zhenhai Rd., Xiamen, 361003 China

**Keywords:** Lung cancer, FDG, PET/CT, Bone biopsy, EGFR, ALK

## Abstract

**Purpose:**

^18^F-FDG PET/CT should be performed before a diagnostic biopsy site is chosen in patients with a high clinical suspicion of aggressive, advanced tumour. The aim of this study was to evaluate the safety and efficacy of ^18^F-FDG PET/CT in guiding biopsy of bone metastases in patients with advanced lung cancer.

**Methods:**

PET/CT-guided percutaneous core biopsies were performed in 51 consecutive patients with suspected lung cancer and ^18^F-FDG-avid bone lesions after whole-body ^18^F-FDG PET/CT scans. Generally, one tissue sample was obtained from each patient. The final diagnoses were established on the basis of the histology results. The histopathological and molecular testing results were systematically evaluated.

**Results:**

A total of 53 samples were obtained for histological examination or molecular testing as a second biopsy was required in two patients in whom the pathological diagnosis was unclear following the first biopsy. The pathological diagnosis and lung cancer classification were confirmed in 48 patients. The epidermal growth factor receptor mutation status was determined in 23 biopsies, and the mutation rate was 30.4 % (7/23). The anaplastic lymphoma kinase mutation status was determined in 19 biopsies, and the mutation rate was 31.6 % (6/19). Two of the 51 biopsies were positive for non-Hodgkin’s lymphoma and one was positive for metastatic renal cell carcinoma. The first-time diagnostic success rate of biopsy was 96.1 % (49/51) and the overall diagnostic success rate and sensitivity were 100 %. All 51 patients were eventually confirmed as having stage IV disease. No serious complications were encountered and the average biopsy time was 30 min.

**Conclusion:**

PET/CT-guided percutaneous biopsy of ^18^F-FDG-avid bone metastases is an effective and safe method that yields a high diagnostic success rate in the evaluation of hypermetabolic bone lesions in patients with suspected advanced lung cancer.

## Introduction

Lung cancer is the primary cause of cancer-related mortality worldwide [1]. When the tumour is limited to the lung, with minimal regional lymph node spread, the most effective therapy is surgery. However, most patients present with locally advanced or metastatic disease and are ineligible for surgical resection. Besides surgical treatment, radiation and chemotherapy, molecularly targeted drugs have emerged as viable therapeutic options in patients with advanced and therefore inoperable cancer. Molecular targets include epidermal growth factor receptor (EGFR), tyrosine kinase inhibitors (TKIs; gefitinib and erlotinib) [2], and anaplastic lymphoma kinase (ALK) TKIs (crizotinib) [3]. Improved pretherapeutic staging, as part of a comprehensive approach to therapy, can limit unnecessary surgical interventions and provide greater benefit to patients.

^18^F-FDG PET/CT has been used to evaluate the extent of disease in cancer patients and to provide more accurate staging [4]. According to the National Comprehensive Cancer Network (NCCN) guidelines for non-small-cell lung cancer (NSCLC), ^18^F-FDG PET can play an important role in the evaluation and accurate staging of NSCLC. However, the guidelines also advocate that each positive PET scan finding for distant disease requires pathological or other radiological confirmation, with biopsy preferred to other methods. Since approximately 30 % to 65 % of patients with metastatic lung carcinoma will develop bone metastases [5], bone biopsy can aid in tumour staging of lung cancer patients with suspected bone metastases.

Although the use of^18^F-FDG PET/CT-guided percutaneous biopsy has been reported in several types of malignant tumour [6, 7], its use for the biopsy of ^18^F-FDG-avid metastatic bone lesions has not been reported in patients with advanced lung cancer. The goal of the present study was to evaluate the safety and efficacy of ^18^F-FDG PET/CT guided biopsy of ^18^F-FDG-avid metastatic bone lesions in patients with advanced lung cancer. The disease in the lung cancer patients was staged on the basis of histopathological examination. In addition, adequate tissue samples from bone biopsies allow more beneficial molecular tests to be performed (i.e. determination of *EGFR* mutation status and *ALK* mutation status), which may identify candidates for individualized treatment and targeted therapy.

## Materials and methods

### Patients

This retrospective evaluation of collected data was approved by the ethics committee of our institution. All patients gave their written informed consent prior to the intervention. Between January 2013 and September 2015, ^18^F-FDG PET/CT-guided bone biopsies of metabolically active lesions were performed in 51 consecutive patients (average age 59.7 years, range 41 – 83 years; 35 men, 16 women) who had undergone whole-body ^18^F-FDG PET/CT for suspected lung cancer. All the patients were biopsied as inpatients and were kept under observation for at least 2 h after the biopsy.

### ^18^F-FDG PET/CT

All patients were asked to fast for at least 4 h prior to undergoing ^18^F-FDG PET/CT. Prior to the scan, all patients received an intravenous injection of 370 – 666 MBq (10 – 18 mCi) of ^18^F-FDG. Data were acquired within 45 min of injection using an integrated PET/CT system (Discovery STE; GE Medical Systems, Milwaukee, WI). The procedure for data acquisition was as follows. CT scanning was performed from the head to the pelvic floor and was matched to the PET section thickness. A PET emission scan was obtained that covered the identical transverse field of view. The acquisition time was 3 min per table position. PET imaging datasets were reconstructed iteratively by applying the CT data for attenuation correction, and coregistered images were displayed on a workstation.

### ^18^F-FDG PET/CT-guided bone biopsy

After the whole-body ^18^F-FDG PET/CT scan the previous day, a board-certified interventional radiologist and nuclear medicine physician performed the bone biopsies in a PET/CT suite dedicated to biopsy procedures. PET/CT-guided bone biopsies were performed using a step-by-step technique. In order to reduce radiation exposure to the medical staff during the biopsy procedures, bone biopsies were scheduled on separate days.

Patients were positioned in a prone or supine position depending on factors such as target location, optimal needle path, and shortest skin-to-target distance. Interventions were performed under aseptic conditions after administration of local anaesthetic with lidocaine.

**Fig. 1:**
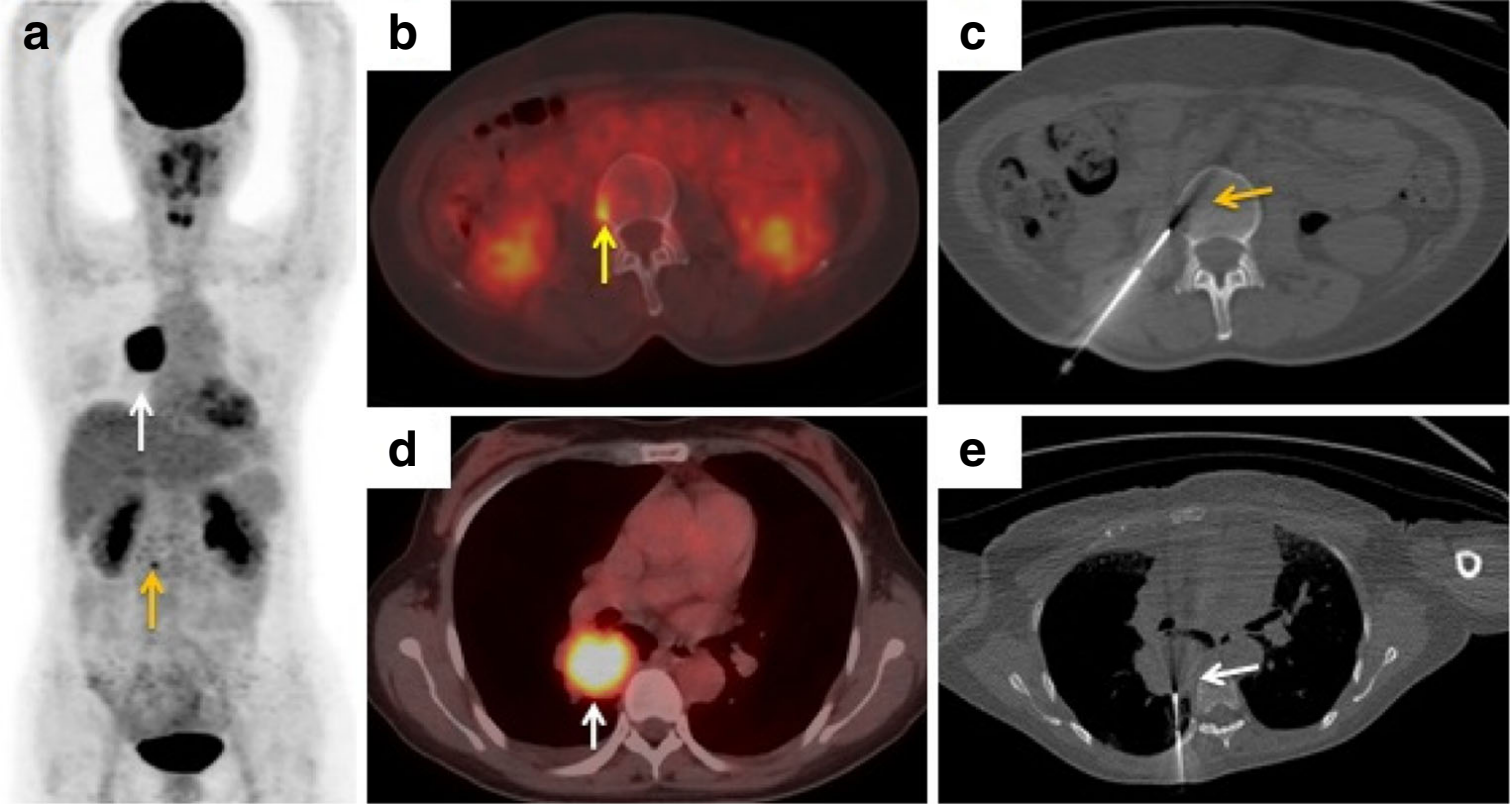
A 58-year-old woman with suspected lung carcinoma and bone metastasis. **a**, **b**, **d** ^18^F-FDG PET/CT imaging (**a** maximum intensity projection image, **b**, **d** axial fusion images) shows uptake (SUVmax 4.6) in a lesion in the third lumbar vertebra on the right (**a**, **b** *yellow arrows*) which is highly suspicious of metastasis, and uptake (SUVmax 11.3) in a lung nodule in the right lower lobe (**a**, **d** *white arrows*). **c** Axial noncontrast CT image (using bone windows) shows the biopsy needle positioned within the lesion (*yellow arrow*). **e** Axial CT image shows the biopsy needle positioned within the lung nodule (*white arrow*). Histological examination confirmed the bone lesion as metastatic poorly differentiated adenocarcinoma and the lung nodule as primary adenosquamous carcinoma, and exon 19 deletion mutation in *EGFR* was detected in both samples

**Fig. 2:**
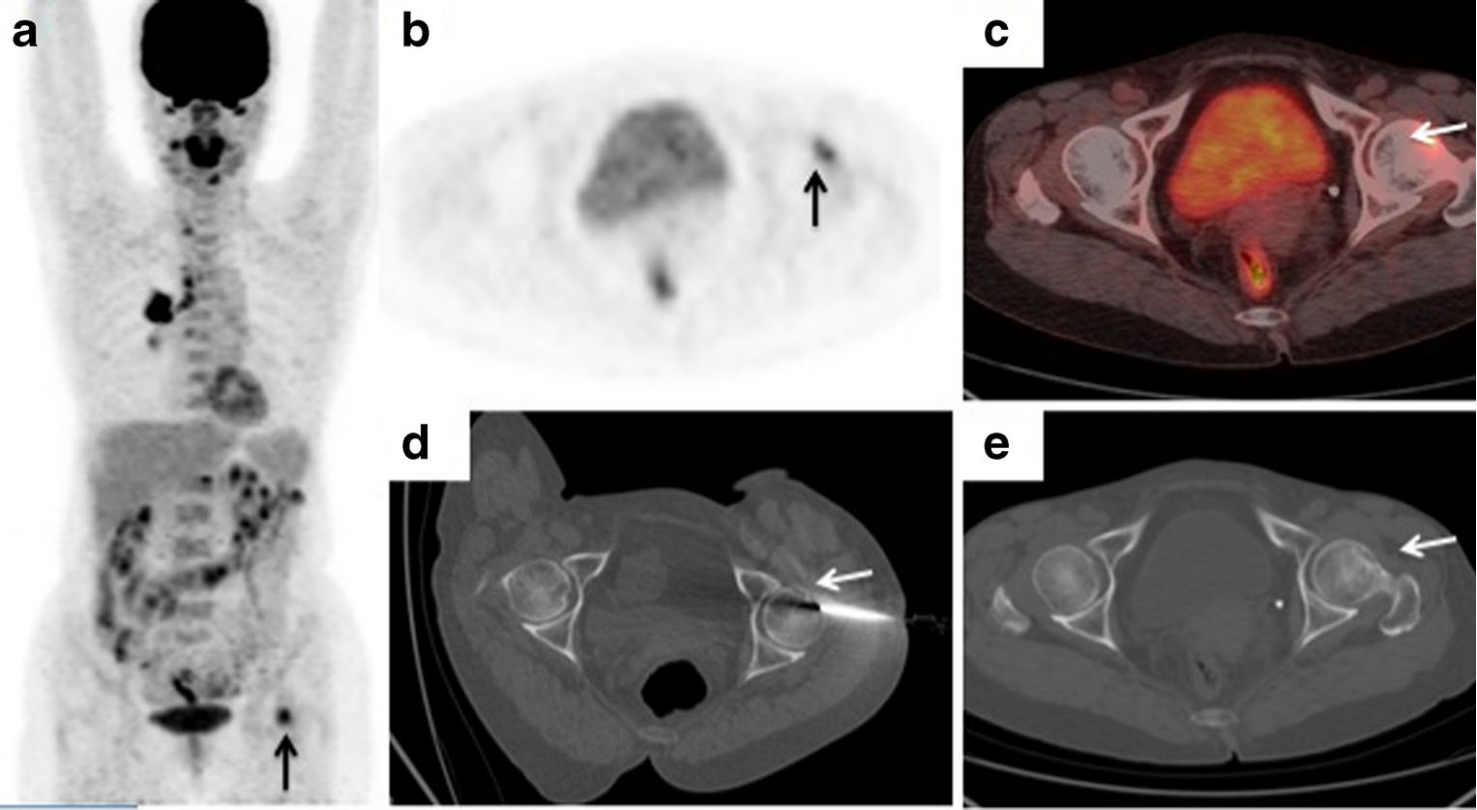
A 61-year-old woman with suspected lung carcinoma with bone metastasis. **a**–**c** ^18^F-FDG PET/CT imaging (**a** maximum intensity projection image. **b** axial PET image. **c** axial PET/CT fusion image) shows uptake (SUVmax 4.8) in a left femoral neck lesion (*arrows*) highly suspicious of metastasis. **d** The CT image shows the biopsy needle positioned within the left femoral neck lesion (*arrow*). **e** The axial noncontrast CT image (using bone windows) shows osteolysis within the corresponding hypermetabolic region (*arrow*). Histological examination confirmed a bone metastasis from lung adenocarcinoma, and exon 19 deletion mutation in *EGFR* was detected

**Fig. 3:**
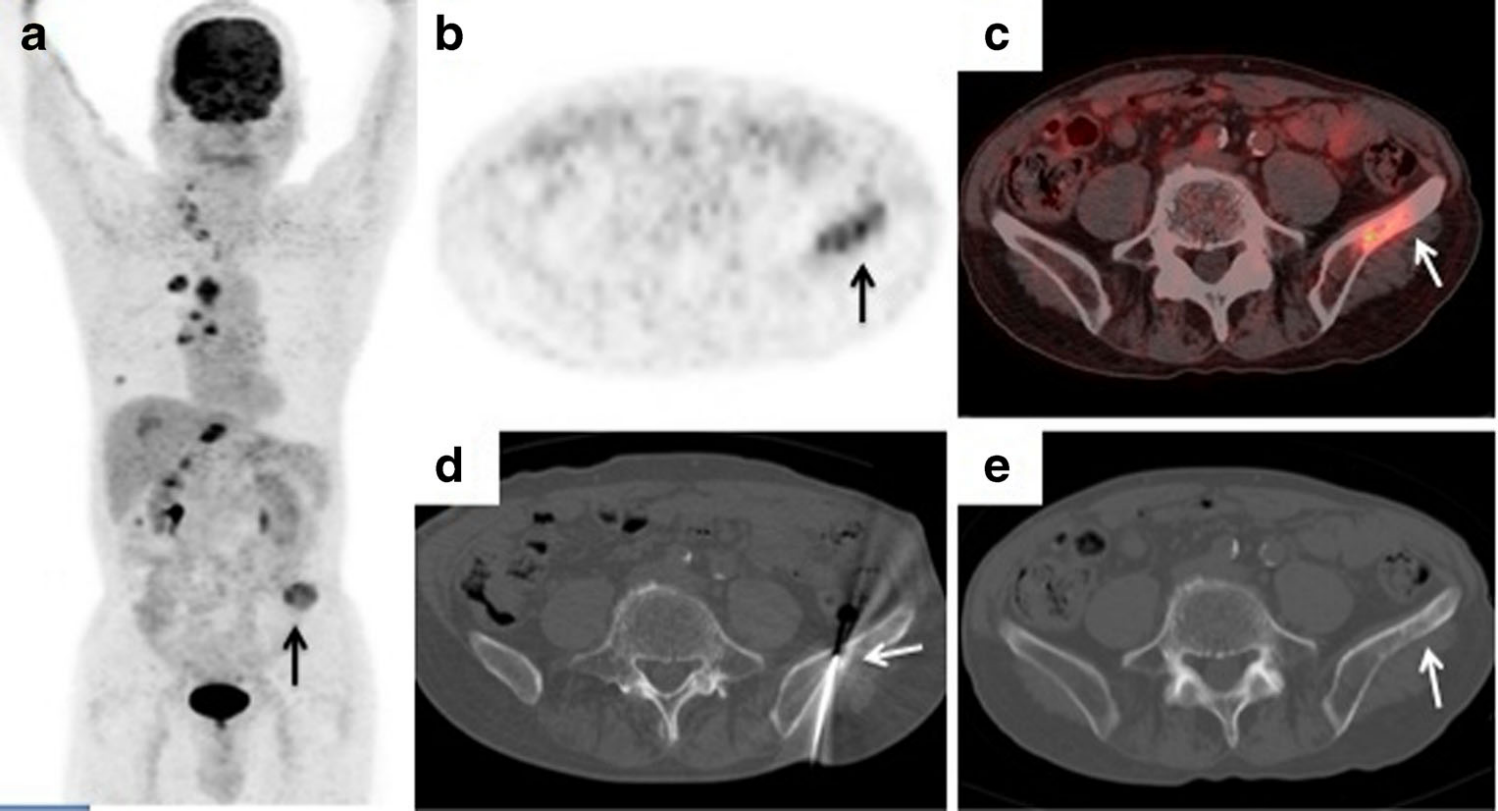
A 76-year-old man with suspected lung carcinoma with bone metastasis. **a**–**c** ^18^F-FDG PET/CT imaging (**a** maximum intensity projection image. **b** axial PET image. **c** axial PET/CT fusion image) shows uptake (SUVmax 6.3) in a lesion in the left ilium (*arrows*) highly suspicious of metastasis. **d** The CT image shows the biopsy needle positioned within the left ilium lesion (*arrow*). **e** The axial noncontrast CT image (using bone windows) shows an osseous abnormality within the corresponding hypermetabolic region (*arrow*). Histological examination confirmed a bone metastasis from poorly differentiated adenocarcinoma of the lung, and exon 21 deletion mutation in *EGFR* was detected

**Fig. 4:**
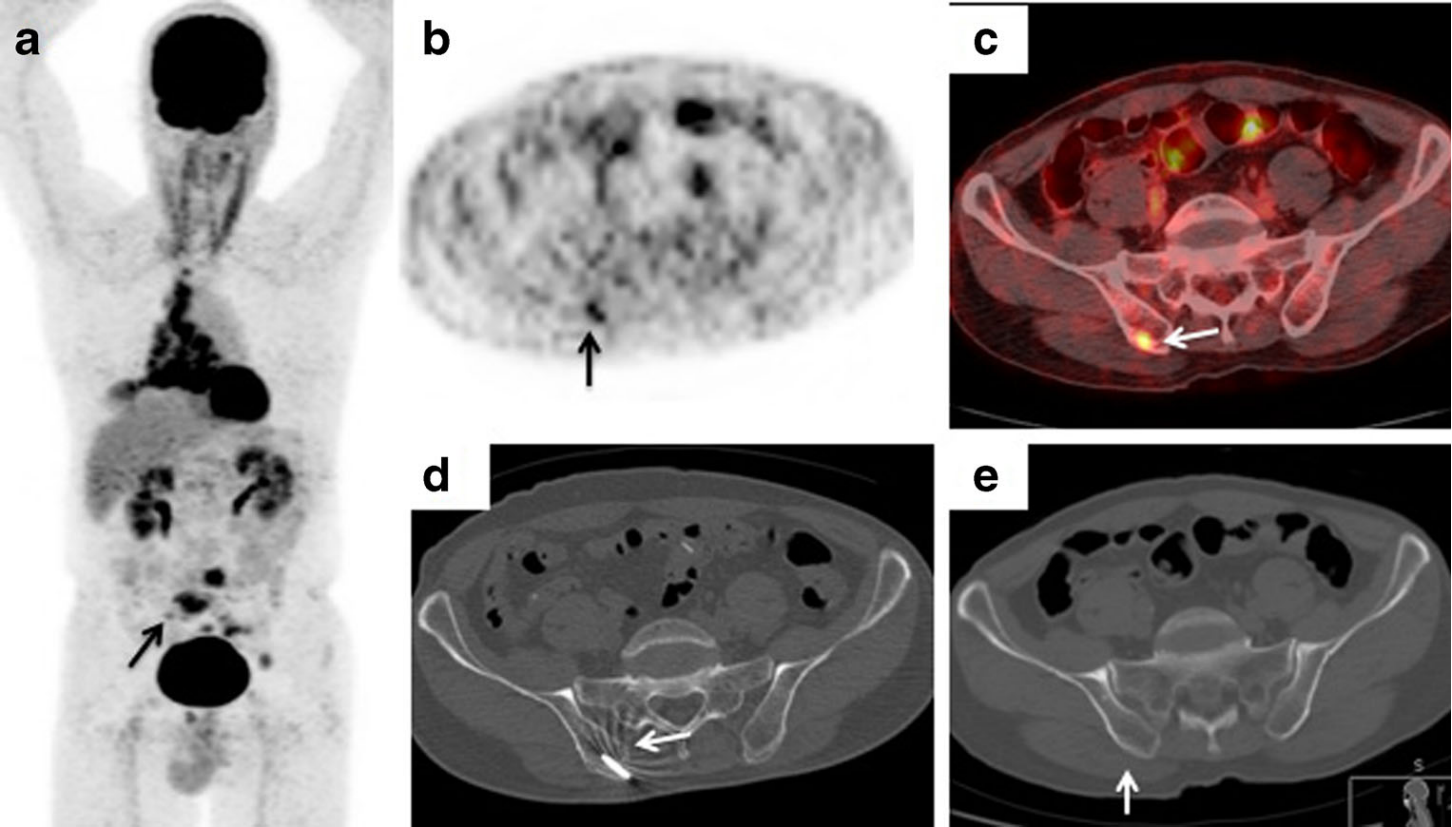
A 62-year-old man with suspected lung carcinoma with multiple bone metastases. **a**–**c** ^18^F-FDG PET/CT imaging (**a** maximum intensity projection image. **b** axial PET image. **c** axial PET/CT fusion image) shows uptake (SUVmax 2.1) within a lesion in the right ilium (*arrows*) highly suspicious of metastasis. **d** The CT image shows the biopsy needle positioned within the right ilium lesion (*arrow*). **e** The axial noncontrast CT image (using bone windows) shows no imaging correlate in the corresponding region (*arrow*). Histological examination confirmed a bone metastasis from small-cell lung cancer of the lung

The needle was introduced in a stepwise manner under fused PET/CT and CT imaging guidance. The bone biopsy needle, either a 16G (Magum, Bard, AZ) or an 11G (BMN-B, SA Medical & Plastic Instruments Co., Ltd, Shanghai, China), was chosen depending on the nature of the lesion (i.e. whether it was an osteoblastic or osteolytic metastasis), and its location and depth. One sample was obtained from each patient, and histopathology and immunohistochemical or molecular diagnosis were performed on each specimen. ^18^F-FDG PET/CT images in representative patients undergoing bone biopsy are shown in Figs 1, 2, 3 and 4.

### Follow-up of the primary lung lesion

All biopsy results were confirmed and a final diagnosis was made in all patients. If the pathological diagnosis based on the bone lesion was not metastatic lung cancer, the pathological results from lung nodules were obtained.

## Results

### ^18^F-FDG PET/CT-guided biopsies

The hypermetabolic bone lesions of interest were successfully targeted and representative tissue samples of the metabolically active sites were obtained in all 51 patients. The biopsy sites are shown in Table 1. A total of 51 samples were obtained for histological examination, and immunohistochemical and/or molecular tests.

**Table 1:** Patient characteristics, biopsy sites and pathological diagnoses

Variable	Value
No. of patients	51
Gender (male/female)	35/16
Age (years), average (range)	59.7 (41 – 83)
Biopsy sites	
Ilium	34
Sacrum	4
Ischium	4
Scapula	2
Rib	2
Femur	2
Clavicle	2
Lumbar vertebra	1
Pathological results	
Lung cancer	48
Adenocarcinoma	36
Squamous carcinoma	3
Small-cell carcinoma	8
Sarcomatoid carcinoma	1
Other	3
Non-Hodgkin’s lymphoma	2
Renal cell carcinoma	1

### Pathological diagnosis from bone biopsy samples

Of the 51 biopsies, 48 were positive for lung cancer (94.1 %; Tables 1 and 2) and 3 were positive for other malignancies (5.9 %; Tables 1 and 3). The 48 lung cancers included 36 adenocarcinomas, 3 squamous carcinomas, 8 small-cell carcinomas, and 1 sarcomatoid carcinoma.The three other malignancies included one metastatic renal cell carcinoma (RCC) and two non-Hodgkin’s lymphoma (NHL). The patient with RCC had undergone tumour resection 3 years prior to this study. The remaining two patients with NHL had no history of malignancy. They both underwent PET/CT-guided lung nodule biopsy, and histology eventually confirmed the primary tumours as NHL.

**Table 2:** Analysis of the 48 PET/CT-guided bone biopsies diagnosed as positive for lung cancer

Biopsy site	No. of biopsies	Biopsy procedure			Diagnosis							
		Success	Complications		H&E + immunohistochemical				Molecular tests			
			Slight pain	Slight bleeding	Adenocarcinoma	Squamous carcinoma	Small-cell carcinoma	Sarcomatoid carcinoma	*ALK* mutation		*EGFR* mutation	
									No. analysed	No. positive	No. analysed	No. positive
Ilium	34	34	29	31	22	3	6	1	19	6	23	7
Sacrum	4	4	4	4	4	0	0	0				
Ischium	4	4	3	4	4	0	0	0				
Scapula	2	2	2	2	2	0	0	0				
Rib	2	2	2	2	0	0	2	0				
Femur	2	2	2	2	2	0	0	0				
Clavicle	1	1	1	1	1	0	0	0				
Lumbar vertebra	1	1	1	1	1	0	0	0				
Total	48	48	42	45	36	3	8	1	19	6	23	7

**Table 3:** Analysis of the three PET/CT-guided bone biopsies with other diagnoses

Diagnosis	Cancer history	Follow-up of lung nodules
Non-Hodgkin’s lymphoma	None	Non-Hodgkin’s lymphoma
Non-Hodgkin’s lymphoma	None	Non-Hodgkin’s lymphoma
Renal cell carcinoma	Renal cell carcinoma	Metastatic renal cell carcinoma

### Success rate of ^18^F-FDG PET/CT-guided bone biopsies

Although only one tissue sample was taken from each patient, there was sufficient tissue to perform haematoxylin and eosin (H&E) staining, immunohistochemical tests, and molecular tests. The first-time diagnostic success rate of ^18^F-FDG PET/CT-guided bone biopsy was 96.1 % (49/51). The overall sensitivity and the diagnostic success rate were 100 %.

### Staging results in all 51 patients

All 51 patients had ^18^F-FDG-avid bone lesions confirmed as cancer metastases. All 51 patients were eventually confirmed as having stage IV cancer.

### Adequacy of tissue samples for molecular tests

Pathological diagnoses were confirmed by histopathology and immunohistochemical tests. In addition, adequate tissue samples made it possible to perform molecular tests. In the 47 biopsies diagnosed with lung cancer, *EGFR* mutation tests were examined in 23 cases and the total mutation rate of bone metastasis was 30.4 % (7/23). *ALK* mutation tests were performed in 19 cases and the mutation rate of bone metastasis was 31.6 % (6/19).

### Complications

Each biopsy procedure took, on average, approximately 30 minutes. Slight bleeding and pain were encountered in almost all patients who underwent biopsy, but no serious complications were noted.

## Discussion

Lung cancer has replaced liver cancer as the primary cause of death among patients with malignancies in China [8]. During the last three decades, the registered lung cancer mortality rate has increased by 464.84 %, which is a heavy burden on patients, health-care professionals, and society [8, 9]. The prognosis of patients with advanced lung cancer is poor, with a 5-year survival rate of 5 % or less. Recent developments in cancer genomics and molecular targeted therapy have caused a major paradigm shift in the management of advanced-stage lung cancer [10]. Precise staging of patients with lung cancer now plays a significant role in determining treatment strategy and prognosis. The accurate definition of tumour stage is a prerequisite for an individualized, risk-adapted therapy for lung cancer. Since ^18^F-FDG PET/CT combines the metabolic information of PET with the anatomic information of CT, it is of significant value in the diagnosis and staging of lung cancer patients [11]. An additional advantage is that it can be used for whole-body scanning with the potential to detect local and distant metastases in a single examination [12].

^18^F-FDG PET/CT has shown excellent diagnostic accuracy in the staging and restaging of a majority of malignant tumours including NSCLC, lymphoma, breast cancer, and liver cancer. When patients with an advanced-stage tumour are accurately staged using^18^F-FDG PET/CT, inappropriate surgery is avoided [13]. However, positive ^18^F-FDG PET/CT findings for distant disease also require pathological or radiological confirmation. Histological examination remains the “gold standard” for definitive diagnosis of lung nodules or lung metastasis. In order to obtain tissue for a histological diagnosis in patients with suspected lung cancer, CT-guided lung biopsy is commonly used [14]. But this invasive procedure is associated with the risk of complications including pain, haemoptysis (1 % to 10 %), pneumothorax (4 % to about 25 %), pneumothorax requiring a chest tube (1 % to about 4 %), air embolism, and atrial fibrillation [14–18]. The elderly with poor pulmonary function are especially at risk of complications. Besides a biopsy of the primary focus, a biopsy of a lung metastasis can usually provide a diagnosis in lung cancer patients. Furthermore, any findings of distant disease can be confirmed by tissue biopsy to ensure accurate staging of the disease [19].

According to the 2015 NCCN guidelines regarding NSCLC, the choice of biopsy should be based on the clinical suspicion of lung cancer, location of the lesion, and patient preferences. Patients suspected of having a solitary site of metastasis should have the disease confirmed in tissue from that site, if feasible. If it is technically difficult or risky to biopsy a particular metastatic site, patients who may have multiple sites of metastatic disease should undergo biopsy of the primary lung lesion or mediastinal lymph nodes. The latest guidelines emphasize the importance of biopsying the site of metastasis as it is preferable to biopsy the pathology that would confer the highest stage (i.e. it is preferable to biopsy a suspected metastasis or mediastinal lymph node rather than a pulmonary lesion [20]).

The optimal puncture site should be selected on the basis of integrated factors, such as target location, optimal needle path and shortest skin-to-target distance. First, when selecting the target location, the most reliably identified focus should be selected. As mentioned above, selecting metastatic tumour rather than the primary lung tumour as the target location may minimize the occurrence of complications. Second, the optimal needle path is very important, because the biopsy process needs avoid nearby blood vessels, the spinal nerve trunk and vital organs. Third, selecting the shortest skin-to-target distance can simplify the biopsy procedure, save time and reduce pain. The aim of all these considerations is to ensure security, feasibility and effectiveness of the procedure. In general, biopsy of FDG-avid bone metastasis was found to meet all these factors simultaneously and it should be considered the best choice for obtaining tumour tissue.

PET imaging is frequently best performed before a diagnostic biopsy site is chosen in patients with a high clinical suspicion of aggressive, advanced-stage tumours. In contrast to a conventional CT-guided biopsy, ^18^F-FDG PET/CT guidance allows targeting of the metabolically active mass or the metabolically active portion of a mass [21]. This novel method can reduce the false-negative rate and is a relatively safe and effective way to obtain a pathological diagnosis. Transoesophageal endobronchial ultrasound-guided transbronchial needle aspiration (EBUS-TBNA) has a high sensitivity and specificity for staging mediastinal and hilar lymph nodes in patients with NSCLC. EBUS-TBNA is of particular value for the biopsy of PET-positive hilar lymph nodes [20, 22].

Since lung cancer patients often have bone metastases, it is important to choose a hypermetabolic bone lesion that is suspected to be a metastasis from lung cancer as the biopsy target after the whole-body ^18^F-FDG PET/CT scan. In the current study, 51 patients underwent PET/CT-guided percutaneous biopsy of ^18^F-FDG-avid metastatic bone lesions. Bone metastases from lung cancer were confirmed pathologically in 48 of the 51 patients, and of the other three patients two were diagnosed as having NHL and one metastatic RCC. Even though two patients underwent a second biopsy since the pathological diagnosis was unclear from the first biopsy, the overall sensitivity was 100 % with a 100 % success rate. In all the patients in this study, the bone biopsy was successful, i.e. the biopsy needle was positioned correctly in the lesion of interest, and a clinically useful histological result was obtained in all 51 patients. Compared with biopsy of pulmonary nodules, bone biopsy avoids hazardous complications such as haemoptysis and pneumothorax. Despite a small amount of bleeding and pain, most patients tolerated the procedure well. Thus, this biopsy method is safe, effective, and feasible as it minimizes the risks while ensuring that an adequate amount of tissue is obtained.

An adequate amount of tissue is required for molecular diagnosis (*EGFR* or *ALK* mutation testing), which plays an important role in the treatment of advanced NSCLC [23]. Conventional methods for obtaining tissue samples such as CT-guided fine needle aspiration, bronchoalveolar aspirate, bronchial alveolar lavage or sampling of pleural effusions provide insufficient material for molecular analysis in a significant proportion of patients [23]. Targeted therapy is of significant value in patients with unresectable lung cancer. EGFR inhibitors, such as erlotinib and gefitinib, are of significant benefit in *EGFR* mutation-positive malignancies, which are primarily adenocarcinomas [24].

In our study, all lung cancer samples were sufficient to perform H&E staining or immunohistochemical tests. *EGFR* mutation status was determined in 23 of the 51 biopsies of bone metastases, and the total mutation rate was 30.4 % (7/23). *ALK* mutation status was determined in 19 biopsies, and the mutation rate was 31.6 % (6/19). Molecular testing of the biopsies from the remaining lung cancer patients was not performed due to lack of funds or the subsequent cost of the targeted treatment. The three other patients had either NHL or metastatic RCC.

In summary, PET/CT-guided biopsy of bone tissue solved the problem of insufficient tissue obtained on biopsy. Our samples from PET/CT-guided biopsy were ample for our needs and enabled us to do more studies with less tissue, i.e. the adequate amount of tissue obtained guaranteed a successful molecular test result.

### Conclusion

This study demonstrated that ^18^F-FDG PET/CT guidance offers the opportunity to precisely target morphologically indeterminate bone lesions for tissue sampling. In lung cancer patients with suspected bone metastases, percutaneous biopsy of ^18^F-FDG-avid metastatic bone lesions is feasible and effective, and can be performed safely with few complications. The use of this biopsy method allows staging and pathological diagnosis. Adequate amounts of tissue can guarantee successful molecular testing, the result of which can be used for individualized treatment and targeted therapy. Thus, percutaneous biopsy of ^18^F-FDG-avid metastatic bone lesions plays an important role in the diagnosis and staging of lung cancer patients with suspected bone metastasis.
